# Quantum Thermodynamics at Strong Coupling: Operator Thermodynamic Functions and Relations

**DOI:** 10.3390/e20060423

**Published:** 2018-05-31

**Authors:** Jen-Tsung Hsiang, Bei-Lok Hu

**Affiliations:** 1 Center for Field Theory and Particle Physics, Department of Physics, Fudan University, Shanghai 200433, China; 2 Maryland Center for Fundamental Physics and Joint Quantum Institute, University of Maryland, College Park, MD 20742-4111, USA

**Keywords:** quantum thermodynamics, strong coupling, operator thermodynamic functions

## Abstract

Identifying or constructing a fine-grained microscopic theory that will emerge under specific conditions to a known macroscopic theory is always a formidable challenge. Thermodynamics is perhaps one of the most powerful theories and best understood examples of emergence in physical sciences, which can be used for understanding the characteristics and mechanisms of emergent processes, both in terms of emergent structures and the emergent laws governing the effective or collective variables. Viewing quantum mechanics as an emergent theory requires a better understanding of all this. In this work we aim at a very modest goal, not quantum mechanics as thermodynamics, not yet, but the thermodynamics of quantum systems, or quantum thermodynamics. We will show why even with this minimal demand, there are many new issues which need be addressed and new rules formulated. The thermodynamics of small quantum many-body systems strongly coupled to a heat bath at low temperatures with non-Markovian behavior contains elements, such as quantum coherence, correlations, entanglement and fluctuations, that are not well recognized in traditional thermodynamics, built on large systems vanishingly weakly coupled to a non-dynamical reservoir. For quantum thermodynamics at strong coupling, one needs to reexamine the meaning of the thermodynamic functions, the viability of the thermodynamic relations and the validity of the thermodynamic laws anew. After a brief motivation, this paper starts with a short overview of the quantum formulation based on Gelin & Thoss and Seifert. We then provide a quantum formulation of Jarzynski’s two representations. We show how to construct the operator thermodynamic potentials, the expectation values of which provide the familiar thermodynamic variables. Constructing the operator thermodynamic functions and verifying or modifying their relations is a necessary first step in the establishment of a viable thermodynamics theory for quantum systems. We mention noteworthy subtleties for quantum thermodynamics at strong coupling, such as in issues related to energy and entropy, and possible ambiguities of their operator forms. We end by indicating some fruitful pathways for further developments.

## 1. Quantum and Thermodynamics—Why?

Why is a paper on this subject matter appearing in this special issue of Entropy? The short answer is the same reason why EmQM appears in this journal Entropy, which is generally considered as treating topics in statistical mechanics: emergence. The long answer, serving as a justification for our dwelling on quantum thermodynamics in the realm of emergent quantum mechanics, is as follows. One way to see quantum mechanics as emergent is by analogy with hydrodynamics and thermodynamics, probably the two best known emergent theories because we know exactly what the collective variables are (thermodynamic functions and relations), the laws they obey (the four laws), how they are related to the basic constituents (molecular dynamics) and the mediating theory from which we can derive both the fundamental and the emergent theories (kinetic theory).

In analogy to emergent gravity [1,2,3,4,5], one of the present authors has championed the thesis of “general relativity as geometro-hydrodynamics” [6,7]. Since Verlinde’s “gravity is entropic force” popularization of Jacobson’s “Einstein equation of state” thesis [8,9,10], gravity as thermodynamics has caught a wider attention in the quantum gravity community. However, a new challenge arises. The question one of us posed for enthusiasts of this theme is the following: Since both gravity and thermodynamics are old subjects established centuries before the advent of quantum mechanics, and both can make sense and stand alone at the classical level without quantum theory, well, what exactly is quantum doing here?—What is the role of quantum in emergent gravity? Do we really need quantum if we view “gravity as thermodynamics”?

### 1.1. Quantum in “Gravity as Thermodynamics”

This question, “Wither the Quantum?”, as Hu calls it, puts the spotlight on quantum, in how it contributes to the emergent phenomena which gives us both gravity and thermodynamics. One answer to this is to also consider quantum mechanics as emergent. For example, in the emergent theories of Adler and ’t Hooft [11,12] probability theory, stochastic and statistical mechanics as a slate play a pivotal role, just as they do for thermodynamics and hydrodynamics arising from molecular dynamics. The same applies to emergent gravity: classical gravity captured by general relativity is an effective theory emergent from some fundamental theories of the basic constituents of spacetime functioning at the sub-Planckian scale. How these basic constituents interact, how their interaction strength varies with energy, how at some specific scale(s) some set(s) of collective variables and the law(s) governing them emerge, and in succession, leading to the physics at the lowest energy as we know it in today’s universe is perhaps just as interesting as the manifestation of the relevant physics at the different scales familiar to us—from molecules to atoms to nucleons to quarks and below. Putting aside gravity for now in this investigation, we wish to see a deeper connection between micro and macro, quantum and thermo.

### 1.2. David Bohm: Quantum in Classical Terms

Here, Bohm’s philosophical influence is evident. His pilot wave theory may not offer a better description or explanation of quantum phenomena, but the view that quantum mechanics is not a fundamental theory any more than a classical wave theory is, provides an inspiration for asking a deeper layer of questions: If we view quantum theory functioning in the capacity as thermodynamics, we should ask: What are the fundamental constituents, the laws governing them, and how quantum mechanics emerges from the sub-structures and theories depicting them (Long before we get to this point, many readers may have raised this objection: This is obviously nonsense: The second law of thermodynamics ostensibly shows the effects of an arrow of time, while quantum mechanics is time-reversal invariant. Well, if the mechanical processes which we can observe are in the underdamped regime where the dissipative effects are not strong enough, they would appear to obey time-reversal symmetry. This is not an outlandish explanation: For most physical systems, in the open system perspective, quantum phenomena in the system appears within the decoherence time which is many orders of magnitude shorter than the relaxation time, as is the case in many well-controlled environments (e.g., cavity QED). Or, if the system is near a nonequilibrium critical point. On this issue, cosmology, despite its seemingly remote bearing, may actually enter in a basic way, in terms of the origin of the arrow of time, and the mere fact that nonequilibrium conditions prevail in an expanding universe.).

### 1.3. Quantum as Thermodynamics?

In quantum thermodynamics, we may not see much in terms of what fundamental theory quantum mechanics emerges from (Adler and ’t Hooft may have their answers: trace dynamics and cellular automaton, for instance, respectively), but even the juxtaposition or crossing of what is traditionally considered as governing the two opposite ends in the macro/micro and classical/quantum spectrum may reveal some deeper meaning in both. Macroscopic quantum phenomena is another such arena. For example, in small quantum systems, at low temperatures, or when the system is strongly coupled to its environment, is there a lower limit to the validity of the laws of thermodynamics, which play such an important role in our understanding of the macro world? Under what conditions will macroscopic entities show quantum phenomena? Is there an upper limit to quantum mechanics governing the meso domain? Is there a limit to quantum commanding the macro world? The above explains the philosophical issues which motivated us to take up a study of quantum thermodynamics. We are also of the opinion that useful philosophical discourses of any subject matter should be based on the hard-core scientific knowledge of that subject, down to all the nitty-gritty details of each important topic that makes up that body of knowledge. Thus we start with the basic demands in the formulation of quantum thermodynamics and try to meet them in a rigorous, no-nonsense way. The specific goal of this paper is to define the operator thermodynamic quantities and spell out their relations for quantum many-body systems in thermal equilibrium.

#### Quantum Thermodynamics

Quantum thermodynamics is a fast developing field, emergent from quantum many body physics and nonequilibrium statistical mechanics. Simply, it is the study of the thermodynamic properties of quantum many-body systems. Quantum now refers not just to the particle spin-statistics (boson vs. fermion) aspects in traditional quantum statistical mechanics, but also includes in the present era the quantum phase aspects, such as quantum coherence, quantum correlations, and quantum entanglement, where quantum information enters. The new challenges arise from several directions not falling under the assumptions of traditional classical thermodynamics: finding the quantum properties of small systems, at zero or very low temperature, strongly coupled to an environment, which could have non-ohmic spectral densities and colored noise, while the system evolves following a non-Markovian dynamics (with memory).

### 1.4. This Work

In this paper we discuss the issues and the technical challenges encountered in the first stage in the construction of a viable theory of quantum thermodynamics, where the system is strongly coupled with a heat bath. We wish to present in a systematic way how to introduce the operator thermodynamic functions and construct their relations. Here, we treat this problem in an equilibrium setting. There are other ways to construct such a theory, such as pursued in the so-called “eigenvalue thermalization” program [13,14,15,16,17,18,19,20,21,22], which treats the system and the environment as a closed system, or along the lines expounded in the fluctuation theorems [23,24,25] where the system under an external drive is allowed to evolve in a nonequilibrium albeit controlled manner [26,27], or in a fully nonequilibrium open quantum system dynamics program (see, e.g., [28,29] and references therein).

## 2. Quantum Thermodynamics at Strong Coupling: Background

### 2.1. New Challenges in Quantum Thermodynamics

Small quantum many-body systems strongly coupled to a heat reservoir at low temperatures are the new focuses of interest for quantum thermodynamics [30,31,32]. Under these hitherto lesser explored conditions, one needs to re-examine the meaning of the thermodynamic functions, the viability of the thermodynamic relations and the validity of the thermodynamic laws anew. Traditional thermodynamics is built on large systems weakly coupled to a reservoir [33], and for quantum systems, only the spin-statistics aspect is studied, as in quantum statistical mechanics, leaving the important factors of quantum coherence, correlations, entanglement and fluctuations as new challenges for quantum thermodynamics. To see the difference strong coupling makes, we take as an example the definition of heat, the energy transferred between the system and the reservoir, for systems strongly coupled to a bath. Esposito et al. [34], for example, show that any heat definition expressed as an energy change in the reservoir energy plus any fraction of the system-reservoir interaction is not an exact differential when evaluated along reversible isothermal transformations, except when that fraction is zero. Even in that latter case the reversible heat divided by temperature, namely entropy, does not satisfy the third law of thermodynamics and diverges in the low temperature limit. For quantum systems, as pointed out by Ankerhold and Pekola [35], in actual measurements, especially for solid state structures, quantum correlations between system and reservoir may be of relevance not only far from but also close to and in thermal equilibrium. Even in the weak coupling regime, this heat flow is substantial at low temperatures and may become comparable to typical predictions for the work based on conventional weak coupling approaches. It further depends sensitively on the non-Markovian features of the reservoir. These observations exemplify the intricacies involved in defining heat for strongly-coupled systems and added complexities for quantum systems, especially at low temperatures.

This incertitude regarding heat translates to ambiguity in the definition of thermodynamic functions and the thermodynamic relations. For example, it was shown [36,37,38,39,40,41] that the expressions for the specific heat derived from the internal energies of a quantum-mechanical harmonic oscillator bilinearly coupled to a harmonic bath calculated by two different approaches can have dramatically different behavior in the low temperature regime. To illustrate this point, Gelin and Thoss [42] compared these two approaches of calculating the internal energy of the system, which give identical results if the system-bath coupling is negligible, but predict significantly differently for finite system-bath coupling. In the first approach, the mean energy of the system given by the expectation value of the system Hamiltonian is evaluated with respect to the total (system + bath) canonical equilibrium distribution. The second approach is based on the partition function of the system, $Z_{s}$, which is postulated to be given as the ratio of the total (system + bath) and the bath partition functions. Gelin and Thoss [42] introduce a bath-induced interaction operator ${\hat{\Delta }}_{s}$, which would account for the effects of finite system-bath coupling and analyze the two approaches for several different systems including several quantum and classical point particles and nonlinear system bath coupling. They found that Approach II leads to very different results from Approach I, their differences exist already within classical mechanics, provided the system-bath interaction is not bilinear and/or the system of interest consists of more than a single particle.

Similar ambiguity appears in the entropy of the quantum system in the same setup. In the first approach, the von Neumann entropy is chosen to be the entropy of the system, while in the second approach, the entropy is given by the derivative of the system’s free energy with respect to the inverse temperature. Both definitions are equivalent in the limit of weak system-bath coupling. However, at finite coupling strength, it has been noticed that [36,37,43,44] the von Neumann entropy may not vanish when the bath temperature is close to zero, even for a simple quantum system that consists of harmonic oscillators. This nonvanishing behavior of the von Neumann entropy is related to the quantum entanglement between the system and the bath [45,46,47,48]. On the other hand, the entropy defined in the second approach of the same system gives an expected vanishing result, consistent with the physical picture described by [49]. These disparities between different choices of the same thermodynamic function of the system can be traced to the degree in which the system-bath interaction enters into or counted as the system.

In a small quantum system, quantum fluctuations of a physical observable of the system can grow to the same order of magnitude as its average value. Thus a description of the thermodynamics of such a system based on the mean value is insufficient. To include the effects of quantum fluctuations of the thermodynamic variables, we need their corresponding quantum operators, from which we can calculate its higher moments. Likewise for quantities related to the quantum correlations and quantum coherence of such a system. Constructing the operator form of a thermodynamic variable is nontrivial and ambiguous at times. Take as an example the internal energy of the system. As is mentioned earlier, it can be given either as the expectation value of the system’s Hamiltonian operator, or as the partial derivative of the (effective) partition function of the system. In the former case, the operator of the internal energy is already chosen but with arbitrariness, while in the latter case, as will be discussed in Section 7.3, our capabilities of identifying the corresponding operator is limited by our knowledge of the thermodynamic variables based on either its expectation or its classical counterpart. Either way there exists non-uniqueness in the introduction of the thermodynamic operator form of the system variables.

Even with a successful extraction and identification of the operator form from the expectation value or the partial derivative of the system’s partition, such a thermodynamic operator for the reduced system in general acts on the Hilbert space of the full composite (system + environment). This is not always a desirable feature because most of the time we are interested in what happens to the system under the influence of the environment, whose details are of no particular concern. A description of the full composite does not easily translate to useful information about the system. Thus it is more preferable to derive operators of the system which solely act on the Hilbert space of the system, without reference to the environment. This is what we try to accomplish here; it is different from what has been reported in the literature.

### 2.2. Goal and Findings of Present Work

In traditional weak coupling thermodynamics, knowing the thermodynamic potentials and their relations enables one to construct a theory of thermodynamics. We believe they are also necessary for the establishment of a theory of thermodynamics for quantum systems. The new challenge is twofold: constructing operator thermodynamic potentials and treating quantum systems strong coupled to their baths. This is our goal in this paper. We shall provide a quantum formulation of Jarzynski’s [27] classical thermodynamics at strong coupling. In contrast to the study of the nonequilibrium dynamics of an open quantum system (called ONEq in [29,50,51]), the combined system + environment studied here is a closed system, called the composite, which is assumed to stay in a global thermal state. In such a configuration, even though the interaction between the system and the bath is non-negligible, the partition function of the composite can be defined. This facilitates the introduction of thermodynamic potentials in a way similar to the traditional vanishing-coupling thermodynamics. We will focus on thermodynamic functions such as internal energy, enthalpy, entropy, and free energies, but exclude the consideration of heat and work in this paper, as quantum work is not well understood and requires a separate treatment.

This paper is organized as follows: In Section 3, we give a quick summary of the familiar thermodynamic relations, which we call traditional or weak-coupling thermodynamics, if only to establish notations. We then consider interacting quantum systems with the help of the Hamiltonian of mean force [52,53,54,55,56,57,58] and discuss some conceptual and technical difficulties we may face when the coupling strength becomes strong. In Section 4, Section 5 and Section 6, we overview the equilibrium quantum formulations of thermodynamics at strong coupling, based on Gelin & Thoss’ and Seifert’s work. In Section 7, we present a equilibrium quantum formulation of Jarzynski’s thermodynamics in the “bare” and “partial molar” representations, respectively in the same setting. We conclude in Section 8 with a short summary, a brief discussion of two issues involved and a suggestion for further developments.

## 3. Thermodynamic Functions, Hamiltonian of Mean Force

We first summarize the familiar traditional thermodynamic relations, if only to establish notations. We then consider interacting quantum systems with the help of the Hamiltonian of mean force (Hamiltonian of mean force is a useful yet not indispensable concept for this purpose. It is useful because in the same representation, the formal expressions associated with it resemble the counterparts in the traditional weak coupling thermodynamics). We outline two quantum formulations of thermodynamic functions and relations; one based on Gelin and Thoss [42] and the other on Seifert [26]. With the abundance of thermodynamic quantities, a word about notations is helpful: quantum expectation values or classical ensemble averages are denoted by math calligraphic, quantum operators associated with the variable *O* will carry an overhat $\hat{O}$.

### 3.1. Traditional (Weak-Coupling) Equilibrium Thermodynamic Relations

The pre-conditions for the traditional weak-coupling thermodynamic theory to be well-defined and operative for a classical or quantum system are very specific despite their wide ranging applicability: (a) A system S of relatively few degrees of freedom is in contact with a thermal bath of a large number or infinite degrees of freedom (We shall consider only heat but no particle transfer here and thus the thermodynamics refers only to canonical, not grand canonical ensembles); (b) the coupling between the system and the bath is vanishingly small; and (c) the system is eternally in a thermal equilibrium state by proxy with the bath which is impervious to any change in the system. In weak-coupling thermodynamics, the bath variables are not dynamical variables (Dynamical variables are those which are determined consistently by the interplay between the system and the bath through their coupled equations of motion); they only provide weak-coupling thermodynamic parameters such as a temperature in canonical ensemble, or, in addition, a chemical potential, in grand canonical ensemble.

The classical thermodynamic relations among the internal energy U, enthalpy H, Helmholtz free energy F and Gibbs free energy G in conjunction with the temperature *T*, entropy S, pressure *P* and volume V are well-known. From the first law, dU=TdS−PdV. With U=U(S,V), we have

(1) $$\begin{array}{cccc} T & ={\left(\frac{\partial U}{\partial S}\right)}_{V}\;,\; & P & =-{\left(\frac{\partial U}{\partial V}\right)}_{S}\;. \end{array}$$

By virtue of the differential form of the internal energy, the enthalpy H=U+PV obeys dH=TdS+VdP. Since it is a function of the entropy and pressure, we can identify

(2) $$\begin{array}{cccc} T & ={\left(\frac{\partial H}{\partial S}\right)}_{P}\;,\; & V & ={\left(\frac{\partial H}{\partial P}\right)}_{S}\;. \end{array}$$

Likewise, for the Helmholtz free energy F=U−TS, we have

(3) $$\begin{array}{ccccccc} dF & =-S\;dT-P\;dV\;, & \mathrm{whence}\;, & S & =-{\left(\frac{\partial F}{\partial T}\right)}_{V}\;, & P & =-{\left(\frac{\partial F}{\partial V}\right)}_{T}\;, \end{array}$$

so the Helmholtz free energy is a function of the temperature and the volume, F=F(T,V). Finally, the Gibbs free energy, defined by G=H−TS, obeys

(4) $$\begin{array}{ccccccc} dG & =-S\;dT+V\;dP\;, & \mathrm{whence}\;, & S & =-{\left(\frac{\partial G}{\partial T}\right)}_{P}\;, & V & ={\left(\frac{\partial G}{\partial P}\right)}_{T}\;. \end{array}$$

Thus G=G(T,P). Many more relations can be derived from these three basic relations. These relations are mutually compatible based on differential calculus.

Next we turn to the weak-coupling thermodynamics of quantum systems (Hereafter, we will choose the units such that the Boltzmann constant $k_{B}=1$ and the reduced Planck constant ℏ=1. In addition, to distinguish them from strong-coupling thermodynamics, all quantities defined in the context of traditional (weak-coupling) thermodynamics are identified with a subscript Θ). The state of a quantum system in contact with a heat bath at temperature $T=\beta^{-1}$ with vanishing coupling is described by the density matrix ${\hat{\rho }}_{s}=e^{-\beta {\hat{H}}_{s}}/Z_{\Theta }$ where $Z_{\Theta }={\mathrm{Tr}}_{s}e^{-\beta {\hat{H}}_{s}}$ is the canonical partition function. Here ${\hat{H}}_{s}$ is the Hamiltonian of the system and is assumed to be independent of the inverse temperature $\beta =T^{-1}$. The notation Tr with a subscript *s* or *b* represents the sum over the states of the system or the bath respectively. The density matrix ${\hat{\rho }}_{s}$ is a time-independent Hermitian operator and is normalized to unity, i.e., ${\mathrm{Tr}}_{s}{\hat{\rho }}_{s}=1$ to ensure unitarity.

The free energy $F_{\Theta }$ of a quantum system in a canonical distribution is $F_{\Theta }=-\beta^{-1}\;\ln Z_{\Theta }$. The quantum expectation value $\langle {\hat{H}}_{s}\rangle$ is identified with the internal energy $U_{\Theta }$ of the quantum system, and can be found by

(5) $$U_{\Theta }=\langle {\hat{H}}_{s}\rangle =\frac{1}{Z_{\Theta }}\;{\mathrm{Tr}}_{s}\left\{{\hat{H}}_{s}\;e^{-\beta {\hat{H}}_{s}}\right\}=-\frac{\partial }{\partial \beta }\ln Z_{\Theta }=F_{\Theta }+\partial_{\beta }F_{\Theta }\;.$$

If we define the entropy $S_{\Theta }$ of the system by

(6) $$S_{\Theta }=\beta^{2}\partial_{\beta }F_{\Theta }\;,$$

then it will be connected with the internal energy by the relation $F_{\Theta }=U_{\Theta }-T\;S_{\Theta }$. These two expressions imply that the entropy of the quantum system can be expressed in terms of the density matrix $S_{\Theta }=-{\mathrm{Tr}}_{s}\left\{{\hat{\rho }}_{s}\ln{\hat{\rho }}_{s}\right\}$, which is the von Neumann entropy. The von Neumann entropy plays an important role in quantum information as a measure of quantum entanglement, and can be used to measure the non-classical correlation in a pure-state system. (Beware of issues at zero temperature as discussed in Section 6.) Here we note that both internal energy and the entropy of the system can be equivalently defined in terms of the expectation values of the quantum operators, or as the derivative of the free energy.

The heat capacity $C_{\Theta }=\partial U_{\Theta }/\partial T=-\beta^{2}\partial_{\beta }U_{\Theta }$ is given by

(7) $$C_{\Theta }=-2\beta^{2}\partial_{\beta }F_{\Theta }-\beta^{3}\partial_{\beta }F_{\Theta }=-\beta \;\partial_{\beta }S_{\Theta }=\beta^{2}\left[\langle {\hat{H}}_{s}^{2}\rangle -{\langle {\hat{H}}_{s}\rangle }^{2}\right]\geq 0\;.$$

It is always semi-positive. Up to this point, under the vanishing system-bath coupling assumption, all the quantum thermodynamic potentials and relations still resemble their classical counterparts.

### 3.2. Quantum System in a Heat Bath with Nonvanishing Coupling

In formulating the quantum thermodynamics at strong coupling, we immediately face some conceptual and technical issues. At strong coupling, the interaction energy between the system and the bath is not negligible, so the total energy cannot be simply divided as the sum of the energies of the system and the bath. This introduces an ambiguity in the definition of, for example, internal energy. We may have more than one way to distribute the interaction energy between the system and the bath. The same ambiguity also arises in the other thermodynamic functions such as enthalpy and entropy, thus affecting the relations among the thermodynamic functions. On the technical side, in the course of formulating quantum thermodynamics, the non-commutative natures of the quantum operators make formidable what used to be straightforward algebraic manipulations in the classical thermodynamics.

Let us illustrate the previous points by an example. Consider in general an interacting quantum system C whose evolution is described by the Hamiltonian ${\hat{H}}_{c}={\hat{H}}_{s}+{\hat{H}}_{i}+{\hat{H}}_{b}$, where ${\hat{H}}_{s},{\hat{H}}_{b}$ are the Hamiltonians of the system S and the bath B, respectively and ${\hat{H}}_{i}$ accounts for the interaction between them. Suppose initially the composite C=S+B is in a global thermal equilibrium state which is stationary, and thus has reversible dynamics, described by the density matrix ${\hat{\rho }}_{c}=e^{-\beta {\hat{H}}_{c}}/Z_{c}$ at the inverse temperature $\beta^{-1}$. The quantity $Z_{c}={\mathrm{Tr}}_{sb}e^{-\beta {\hat{H}}_{c}}$ is the partition function of the composite for the global thermal state.

In the case of vanishing coupling between the system and the bath, we may approximate the total Hamiltonian ${\hat{H}}_{c}$ to the leading order by ${\hat{H}}_{c}≃{\hat{H}}_{s}+{\hat{H}}_{b}$. Since $[{\hat{H}}_{s},{\hat{H}}_{b}]=0$, we notice that

(8) $$\frac{1}{Z_{b}}\;{\mathrm{Tr}}_{b}e^{-\beta {\hat{H}}_{c}}≃\frac{1}{Z_{b}}\;{\mathrm{Tr}}_{b}\left\{e^{-\beta {\hat{H}}_{s}}e^{-\beta {\hat{H}}_{b}}\right\}=e^{-\beta {\hat{H}}_{s}}\;,$$

with the partition function of the free bath being given by $Z_{b}={\mathrm{Tr}}_{b}e^{-\beta {\hat{H}}_{b}}$. Equation (8) implies that the reduced state ${\hat{\rho }}_{r}={\mathrm{Tr}}_{b}{\hat{\rho }}_{c}$, which is also stationary, will assume a canonical form

(9) $$\begin{array}{ccccc} \rho_{r} & =\frac{e^{-\beta {\hat{H}}_{s}}}{Z_{s}}=\frac{1}{Z_{c}}{\mathrm{Tr}}_{b}e^{-\beta {\hat{H}}_{c}}\;, & \mathrm{with} & Z_{s} & ={\mathrm{Tr}}_{s}e^{-\beta {\hat{H}}_{s}}\;, \end{array}$$

that is, $Z_{c}≃Z_{s}Z_{b}$ in the limit of vanishing system-bath coupling. In addition, (9) ensures the proper normalization condition ${\mathrm{Tr}}_{s}\rho_{r}=1$. Thus in the weak limit of the system-bath interaction, the reduced density matrix of the interacting composite system in the global thermal state will take the canonical form, hence it to some degree justifies the choice of the system state in the context of quantum thermodynamics in the textbooks [59,60]. Hereafter we will denote the reduced density matrix of the system by ${\hat{\rho }}_{s}$.

When the interaction between the system and the bath cannot be neglected, the righthand side of (8) no longer holds. In addition, non-commutating nature among the operators ${\hat{H}}_{s}$, ${\hat{H}}_{i}$ and ${\hat{H}}_{b}$ prevents us from writing $e^{-\beta ({\hat{H}}_{s}+{\hat{H}}_{i}+{\hat{H}}_{b})}\neq e^{-\beta ({\hat{H}}_{s}+{\hat{H}}_{b})}e^{-\beta {\hat{H}}_{i}}$, due to $[{\hat{H}}_{s},{\hat{H}}_{i}]\neq 0$ and $[{\hat{H}}_{b},{\hat{H}}_{i}]\neq 0$ in general. In fact, according to the Baker-Campbell-Haussdorff (BCH) formula, the previous decomposition will have the form

(10) $$\begin{array}{cc} e^{-\beta ({\hat{H}}_{s}+{\hat{H}}_{b})}e^{-\beta {\hat{H}}_{i}} & =\exp\left\{-\beta \left({\hat{H}}_{s}+{\hat{H}}_{i}+{\hat{H}}_{b}\right)+\frac{\beta^{2}}{2!}\;\left[{\hat{H}}_{s}+{\hat{H}}_{b},{\hat{H}}_{i}\right]\right.\\ & \;\;-\left.\frac{\beta^{3}}{3!}\;\left(\frac{1}{2}\;\left[\left[{\hat{H}}_{s}+{\hat{H}}_{b},{\hat{H}}_{i}\right],{\hat{H}}_{i}\right]+\frac{1}{2}\;\left[{\hat{H}}_{s}+{\hat{H}}_{b},\left[{\hat{H}}_{s}+{\hat{H}}_{b},{\hat{H}}_{i}\right]\right]\right)+\cdots \right\}\;. \end{array}$$

The exponent on the righthand side typically contains an infinite number of terms. This makes algebraic manipulation of the strongly interacting system rather complicated, in contrast to its classical or quantum weak-coupling counterpart.

### 3.3. Hamiltonian of Mean Force

To account for non-vanishing interactions (in this paper, we apply the Hamiltonian of mean force to a quantum system in the global thermal state setup without any time-dependent driving force. See [53,54,55] for its use in nonequilibrium systems at strong coupling), one can introduce the Hamiltonian of mean force $H_{s}^{*}$ for the system defined by [56,57,58]

(11) $$e^{-\beta {\hat{H}}_{s}^{*}}\equiv \frac{1}{Z_{b}}\;{\mathrm{Tr}}_{b}e^{-\beta {\hat{H}}_{c}}\;.$$

It depends only on the system operator but has included all the influences from the bath. In the limit ${\hat{H}}_{i}$ is negligible H^s∗≃H^s∗; otherwise, in general H^s∗≠H^s∗. The corresponding partition function $Z^{*}$ is then given by $Z^{*}={\mathrm{Tr}}_{s}e^{-\beta {\hat{H}}_{s}^{*}}=Z_{b}^{-1}\;{\mathrm{Tr}}_{sb}e^{-\beta {\hat{H}}_{c}}=Z_{c}/Z_{b}$.

If one followed the procedure of traditional weak-coupling thermodynamics to define the free energy as $F=-\beta^{-1}\ln Z$, then the total free energy $F_{c}$ of the composite system can be given by a simple additive expression $F_{c}=F^{*}+F_{b}$, with $F^{*}=-\beta^{-1}\ln Z^{*}$ and $F_{b}=-\beta^{-1}\ln Z_{b}$. In addition, one can write the reduced density matrix ${\hat{\rho }}_{s}$ in a form similar to (9), with the replacement of ${\hat{H}}_{s}$ by ${\hat{H}}_{s}^{*}$,

(12) $${\hat{\rho }}_{s}=\frac{1}{Z_{c}}\;{\mathrm{Tr}}_{b}e^{-\beta {\hat{H}}_{c}}=\frac{e^{-\beta {\hat{H}}_{s}^{*}}}{Z^{*}}\;,$$

in the hope that the conventional procedures of weak-coupling thermodynamics will follow. However, in Section 7 or in [42], we see that at strong coupling even though we already have the (reduced) density matrix and the free energy of the system, we can introduce two different sets of thermodynamic potentials for the system. The thermodynamic potentials in each set are mathematically self-consistent, but they are not compatible with their counterparts in the other set, in contrast to the weak coupling limit, where both definitions are equivalent.

Two earlier approaches to introduce the thermodynamic potentials in a strongly interacting system had been proposed by Gelin and Thoss [42], and by Seifert [26]. We shall summarize the approach I in Gelin and Thoss’ work below and present a more detailed quantum formulation of Seifert’s approach following, both for a configuration that the composite is initially in a global thermal state without any external force. A recent proposal by Jarzynski [27] for classical systems will be formulated quantum-mechanically in the same setting in Section 7.

## 4. Quantum Formulation of Gelin and Thoss’ Thermodynamics at Strong Coupling

The first approach, based on Approach I of Gelin & Thoss [42], is rather intuitive, because their definitions of the internal energy and the entropy are the familiar ones in traditional thermodynamics. They define the internal energy $U_{s}$ of the (reduced) system by the quantum expectation value of the system Hamiltonian operator alone, $U_{s}={\mathrm{Tr}}_{s}\left\{{\hat{\rho }}_{s}\;{\hat{H}}_{s}\right\}$, and choose the entropy to be the von Neumann (vN) entropy $S_{s}=S_{vN}=-{\mathrm{Tr}}_{s}\left\{{\hat{\rho }}_{s}\ln{\hat{\rho }}_{s}\right\}$. These are borrowed from the corresponding definitions in weak-coupling thermodynamics.

They write the same reduced density matrix (9) in a slightly different representation to highlight the difference from the weak-coupling thermodynamics case,

(13) $$\begin{array}{ccccc} {\hat{\rho }}_{s} & =\frac{1}{Z_{c}}\;e^{-\beta ({\hat{H}}_{s}+{\hat{\Delta }}_{s})}=e^{-\beta ({\hat{H}}_{s}+{\hat{\Delta }}_{s}-F_{c})}\;, & \mathrm{with} & Z_{c} & ={\mathrm{Tr}}_{s}e^{-\beta ({\hat{H}}_{s}+{\hat{\Delta }}_{s})}=e^{-\beta F_{c}} \end{array}$$

where ${\hat{\Delta }}_{s}$ depends only on the system variables but includes all of the influence from the bath from their interaction. Comparing this with (11), we note that ${\hat{\Delta }}_{s}$ is formally related to the Hamiltonian of mean force by ${\hat{\Delta }}_{s}={\hat{H}}_{s}^{*}-{\hat{H}}_{s}+F_{b}$. Finally, they let the partition function of the system take on the value $Z_{c}$, which is distinct from $Z^{*}$. Thus the corresponding free energy will be given by $F_{c}$ which contains all the contributions from the composite C.

Although in this approach the definitions of internal energy and entropy of the system are quite intuitive, these two thermodynamic quantities do not enjoy simple relations with the partition function $Z_{c}$, as in (5). From (13), we can show (Here some discretion is advised in taking the derivative with respect to β because in general an operator will not commute with its own derivative. See details in Appendix A)

(14) $$\begin{array}{cc} -\frac{\partial }{\partial \beta }\;\ln Z_{c}=-\frac{1}{Z_{c}}\frac{\partial }{\partial \beta }{\mathrm{Tr}}_{s}e^{-\beta ({\hat{H}}_{s}+{\hat{\Delta }}_{s})} & =\langle {\hat{H}}_{s}\rangle +\langle {\hat{\Delta }}_{s}\rangle +\beta \;\langle \partial_{\beta }{\hat{\Delta }}_{s}\rangle \neq U_{s}\;, \end{array}$$

with the corresponding free energy $F_{c}=U_{s}+\langle {\hat{\Delta }}_{s}\rangle +\beta \;\langle \partial_{\beta }{\hat{\Delta }}_{s}\rangle -\beta \;\partial_{\beta }F_{c}$. Here 〈⋯〉 represents the expectation value taken with respect to the density matrix ${\hat{\rho }}_{c}$ of the composite. For a system operator ${\hat{O}}_{s}$, this definition yields an expectation value equal to that with respect to the reduced density matrix ${\hat{\rho }}_{s}$, namely,

(15) $${\langle {\hat{O}}_{s}\rangle }_{s}={\mathrm{Tr}}_{s}\left\{{\hat{\rho }}_{s}\;{\hat{O}}_{s}\right\}={\mathrm{Tr}}_{sb}\left\{{\hat{\rho }}_{c}\;{\hat{O}}_{s}\right\}=\langle {\hat{O}}_{s}\rangle \;.$$

Likewise, the von Neumann entropy $S_{vN}$ can be expressed in terms of the free energy $F_{c}$ by

(16) $$S_{vN}=\beta^{2}\partial_{\beta }F_{c}-\beta^{2}\langle \partial_{\beta }{\hat{\Delta }}_{s}\rangle \;,$$

which does not resemble the traditional form in (3). Additionally, we observe the entropy so defined is not additive, that is,

(17) $$S_{vN}+S_{b}=-{\mathrm{Tr}}_{s}\left\{{\hat{\rho }}_{s}\ln{\hat{\rho }}_{s}\right\}-{\mathrm{Tr}}_{b}\left\{{\hat{\rho }}_{s}\ln{\hat{\rho }}_{b}\right\}\neq -{\mathrm{Tr}}_{sb}\left\{{\hat{\rho }}_{c}\ln{\hat{\rho }}_{c}\right\}=S_{c}\;.$$

Here $S_{c}$ and $S_{b}$ are the von Neumann entropies of the composite and the free bath, respectively. Note that the ${\hat{\rho }}_{b}$ in this formulation is the density matrix of the free bath, not the reduced density matrix of the bath, namely, ${\hat{\rho }}_{b}\neq {\mathrm{Tr}}_{s}{\hat{\rho }}_{c}$. The reduced density matrix of the bath will contain an additional overlap with the system owing to their coupling.

When the internal energy of the system given by the expectation value of the system Hamiltonian operator ${\hat{H}}_{s}$, the specific heat $C_{s}$ will take the form,

(18) $$\begin{array}{cc} C_{s}=-\beta^{2}\partial_{\beta }\langle {\hat{H}}_{s}\rangle & =-\beta \;\partial_{\beta }S_{vN}-\beta^{2}\left[\langle \partial_{\beta }{\hat{\Delta }}_{s}\rangle -\partial_{\beta }\langle {\hat{\Delta }}_{s}\rangle \right]\;. \end{array}$$

In general $\langle \partial_{\beta }{\hat{\Delta }}_{s}\rangle \neq \partial_{\beta }\langle {\hat{\Delta }}_{s}\rangle$ since the reduced density matrix ${\hat{\rho }}_{s}$ also has a temperature dependence. We thus see in this case the heat capacity cannot be directly given as the derivative of the (von Neumann) entropy with respect to β, as in (7).

In short, in this formulation, the thermodynamic potentials of the system are defined in a direct and intuitive way. They introduce an operator ${\hat{\Delta }}_{s}$ to highlight the foreseen ambiguity when the system is strongly coupled with the bath. Formally we see that ${\hat{\Delta }}_{s}=-\beta^{-1}\ln{\mathrm{Tr}}_{b}e^{-\beta ({\hat{H}}_{s}+{\hat{H}}_{i}+{\hat{H}}_{b})}-{\hat{H}}_{s}$. In the limit of weak coupling, ${\hat{H}}_{i}\approx 0$, the operator ${\hat{\Delta }}_{s}$ reduces to

(19) $$\begin{array}{cc} {\hat{\Delta }}_{s} & \approx -\beta^{-1}\ln{\mathrm{Tr}}_{b}e^{-\beta ({\hat{H}}_{s}+{\hat{H}}_{b})}-{\hat{H}}_{s}=-\beta^{-1}\ln Z_{b}\;. \end{array}$$

Hence in this limit, ${\hat{\Delta }}_{s}$ reduces to a *c*-number and plays the role of the free energy $F_{b}$ of the free bath. Observe ${\hat{\Delta }}_{s}\approx F_{b}$ in the weak coupling limit annuls the expression in the square brackets in (18) and restores the traditional relation (7) between the heat capacity and the entropy. However, even in the weak coupling limit, the internal energy still cannot be given by (5). The disparity lies in the identification of $Z_{c}$ as the partition function of the system. As is clearly seen from (14), in the weak coupling limit, we have

(20) $$\begin{array}{c} -\frac{\partial }{\partial \beta }\;\ln Z_{c}\approx -\frac{1}{Z_{c}}\frac{\partial }{\partial \beta }{\mathrm{Tr}}_{s}\left\{e^{-\beta {\hat{H}}_{s}}Z_{b}\right\}={\langle {\hat{H}}_{s}\rangle }_{s}+{\langle {\hat{H}}_{b}\rangle }_{b}\;. \end{array}$$

This implies that $Z_{c}$ is not a good candidate for the partition function of the system. A more suitable option would be $Z_{c}/Z_{b}$.

## 5. Quantum Formulation of Seifert’s Thermodynamics at Strong Coupling

If we literally follow (11) and identify ${\hat{H}}_{s}^{*}$ as the effective Hamiltonian operator of the (reduced) system, we will nominally interpret that the reduced system assumes a canonical distribution. Thus it is natural to identify $Z^{*}$ as the partition function associated with the reduced state of the system.

Suppose we maintain the thermodynamic relations regardless of the coupling strength between the system and the bath. From (5) to (6), we will arrive at expressions of the internal energy and entropy of the system. This is essentially Seifert’s approach [26] to the thermodynamics at strong coupling. Here we will present the quantum-mechanical version of it for a equilibrium configuration without the external drive, that is, λ=0 in Seifert’s notion.

First, from (11), we have the explicit form of the Hamiltonian of mean force ${\hat{H}}_{s}^{*}$

(21) $$\begin{array}{c} {\hat{H}}_{s}^{*}=-\beta^{-1}\ln{\mathrm{Tr}}_{b}\left\{\exp\left[-\beta {\hat{H}}_{s}-\beta {\hat{H}}_{i}-\beta \left({\hat{H}}_{b}-F_{b}\right)\right]\right\}\;. \end{array}$$

This is the operator form of $H(\xi_{s},\lambda )$ in Equation (5) of [26]. Noting the non-commutative characters of the operators. Since $[{\hat{H}}_{s},{\hat{H}}_{i}]\neq 0$,

(22) $$e^{-\beta ({\hat{H}}_{s}+{\hat{H}}_{i}+{\hat{H}}_{b})}↛e^{-\beta {\hat{H}}_{s}}e^{-\beta ({\hat{H}}_{i}+{\hat{H}}_{b})}\;.$$

If one prefers factoring out $e^{-\beta {\hat{H}}_{s}}$ from $e^{-\beta ({\hat{H}}_{s}+{\hat{H}}_{i}+{\hat{H}}_{b})}$, one can use the Baker-Campbell-Haussdorff formula, outlined in Appendix A, to expand out the operator products to a certain order commensurate with a specified degree of accuracy.

Second, it is readily seen that $p^{\mathrm{eq}}\left(\xi_{s}|\lambda =0\right)$ in Equation (4) of [26] is the reduced density matrix ${\hat{\rho }}_{s}$ of the system (12). The (Helmholtz) free energy F in Seifert’s Equation (7) is exactly the free energy of the reduced system $F^{*}$ in Section 3.3.

With these identifications, it is easier to find the rest of the physical quantities in Seifert’s strong coupling thermodynamics for the equilibrium configuration. We now proceed to derive the entropy and the internal energy, i.e., Equations (8) and (9) of [26], for quantum systems in his framework in the equilibrium setting.

In general, an operator does not commute with its derivative, so taking the derivative of an operator-valued function or performing integration by parts on an operator-valued function can be nontrivial. Their subtleties are discussed in Appendix A, where we show that the derivative of an operator function is in general realized by its Taylor’s expansion in a symmetrized form (A5). However, when such a form appears in the trace, the cyclic property of the trace allow us to manipulate the derivative of a operator-valued function as that of a *c*-number function thus sidestepping the symmetrized ordering challenge. Hence from the thermodynamic relation (6), we have

(23) $$\begin{array}{cc} S_{s} & =-\beta F^{*}+\beta \;{\mathrm{Tr}}_{s}\left\{{\hat{\rho }}_{s}\left({\hat{H}}_{s}^{*}+\beta \;\partial_{\beta }{\hat{H}}_{s}^{*}\right)\right\}\;. \end{array}$$

Here we recall that even though the operators ${\hat{H}}_{s}^{*}$ and $\partial_{\beta }{\hat{H}}_{s}^{*}$ in general do not commute, the trace operation allowing for cyclic permutations of the operator products eases the difficulties in their manipulation. Since (12) implies the operator identity $\beta {\hat{H}}_{s}^{*}=\beta F^{*}-\ln{\hat{\rho }}_{s}$, we can recast (23) to

(24) $$\begin{array}{c} S_{s}={\mathrm{Tr}}_{s}\left\{{\hat{\rho }}_{s}\left(-\ln\rho_{s}+\beta^{2}\partial_{\beta }{\hat{H}}_{s}^{*}\right)\right\}=S_{vN}+\beta^{2}\langle \partial_{\beta }H_{s}^{*}\rangle \neq -{\mathrm{Tr}}_{s}\left\{{\hat{\rho }}_{s}\ln{\hat{\rho }}_{s}\right\}=S_{vN}\;. \end{array}$$

This is the quantum counterpart of Seifert’s entropy, Equation (8) of [26]. This entropy is often called the “thermodynamic” entropy in the literature. Note that it is not equal to the von Neumann (“statistical”) entropy $S_{vN}$ of the system.

The internal energy can be given by the thermodynamic relation

(25) $$U_{s}=F^{*}+\beta^{-1}S_{s}\;.$$

Thus from (23), we obtain,

(26) $$\begin{array}{c} U_{s}={\mathrm{Tr}}_{s}\left\{{\hat{\rho }}_{s}\left({\hat{H}}_{s}^{*}+\beta \;\partial_{\beta }{\hat{H}}_{s}^{*}\right)\right\}=\langle {\hat{H}}_{s}^{*}\rangle +\beta \;\langle \partial_{\beta }{\hat{H}}_{s}^{*}\rangle \neq \langle {\hat{H}}_{s}^{*}\rangle \;. \end{array}$$

This deviation results from the fact that ${\hat{H}}_{s}^{*}$, introduced in (11) may depend on β. When we take this into consideration, we can verify that the internal energy can also be consistently given by Equation (5)

(27) $$\begin{array}{cc} -\frac{\partial }{\partial \beta }\;\ln Z^{*} & =\frac{1}{Z^{*}}{\mathrm{Tr}}_{s}\left\{\left({\hat{H}}_{s}^{*}+\beta \;\partial_{\beta }{\hat{H}}_{s}^{*}\right)\;e^{-\beta {\hat{H}}_{s}^{*}}\right\}=U_{s}\;. \end{array}$$

In fact, we can show, by recognizing $Z^{*}=Z_{c}/Z_{b}$, that

(28) $$U_{s}=\langle {\hat{H}}_{s}\rangle +\left[\langle {\hat{H}}_{i}\rangle +\langle {\hat{H}}_{b}\rangle -{\langle {\hat{H}}_{b}\rangle }_{b}\right]\neq \langle {\hat{H}}_{s}\rangle \;,$$

with ${\langle {\hat{H}}_{b}\rangle }_{b}\equiv {\mathrm{Tr}}_{b}\left\{{\hat{\rho }}_{b}\;{\hat{H}}_{b}\right\}$, $\langle {\hat{H}}_{b}\rangle \equiv {\mathrm{Tr}}_{sb}\left\{{\hat{\rho }}_{c}\;{\hat{H}}_{b}\right\}$, and $\langle {\hat{H}}_{s}\rangle \equiv {\mathrm{Tr}}_{sb}\left\{{\hat{\rho }}_{c}\;{\hat{H}}_{s}\right\}={\langle {\hat{H}}_{s}\rangle }_{s}$. Equation (28) implies that the internal energy, defined by (27), accommodates more than mere ${\langle {\hat{H}}_{s}\rangle }_{s}$. The additional pieces contain contributions from the bath and the interaction. In particular, when the coupling between the system and the bath is not negligible, we have $\langle {\hat{H}}_{b}\rangle \neq {\langle {\hat{H}}_{b}\rangle }_{b}$ in general. As a matter of fact, even the internal energy defined by the expectation value of the system Hamiltonian operator in approach I of Gelin & Thoss’ work also encompasses influence from the bath because the reduced density matrix ${\hat{\rho }}_{s}$ includes all the effects of the bath on the system.

Hitherto, we have encountered three possible definitions of internal energies, namely, $\langle {\hat{H}}_{s}\rangle$, $\langle {\hat{H}}_{s}^{*}\rangle$, and $U_{s}$. As can be clearly seen from (26) and (28), they essentially differ by the amount of the bath and the interaction energy which are counted toward the system energy. This ambiguity arises from strong coupling between the system and the bath. When the system-bath interaction is negligibly small, we have $\langle {\hat{H}}_{i}\rangle \approx 0$, and since in this limit, the full density matrix of the composite is approximately given by the product of that of the system and the bath, we arrive at $\langle {\hat{H}}_{b}\rangle \approx {\langle {\hat{H}}_{b}\rangle }_{b}$, and these three energies become equivalent.

To explicate the physical content of $\langle {\hat{H}}_{s}^{*}\rangle$, from (12) we can write $\langle {\hat{H}}_{s}^{*}\rangle$ as

(29) $$\begin{array}{ccccc} \langle {\hat{H}}_{s}^{*}\rangle & =-\frac{1}{\beta }\;{\mathrm{Tr}}_{s}\left\{{\hat{\rho }}_{s}\ln{\hat{\rho }}_{s}\right\}+F^{*}=\beta^{-1}S_{vN}+F^{*}\;, & \mathrm{or} & F^{*} & =\langle {\hat{H}}_{s}^{*}\rangle -\beta^{-1}\;S_{vN}\;. \end{array}$$

This offers an interesting comparison with (25), where $F^{*}=U_{s}-\beta^{-1}S_{s}$. It may appear that we can replace the pair $\left(U_{s},S_{s}\right)$ by another pair $\left(\langle {\hat{H}}_{s}^{*}\rangle ,S_{vN}\right)$, leaving $F^{*}$ unchanged, thus suggesting an alternative definition of internal energy by $\langle {\hat{H}}_{s}^{*}\rangle$ and that of entropy by $S_{vN}$. However, in so doing, the new energy and entropy will not satisfy a simple thermodynamic relation like (5) and (6). This is a good sign, as it is an indication that certain internal consistency exists in the choice of the thermodynamic variables.

We now investigate the differences between the two definitions of entropy. From (24), we obtain

(30) $$\begin{array}{c} T\left(S_{s}-S_{vN}\right)={\mathrm{Tr}}_{s}\left\{{\hat{\rho }}_{s}\left(\beta \;\partial_{\beta }{\hat{H}}_{s}^{*}\right)\right\}=\beta \;\partial_{\beta }{\mathrm{Tr}}_{s}\left\{{\hat{\rho }}_{s}\;{\hat{H}}_{s}^{*}\right\}-\beta {\mathrm{Tr}}_{s}\left\{\left(\partial_{\beta }{\hat{\rho }}_{s}\right){\hat{H}}_{s}^{*}\right\}\;. \end{array}$$

The factor $\partial_{\beta }{\hat{\rho }}_{s}$ can be written as $\partial_{\beta }{\hat{\rho }}_{s}=\langle {\hat{H}}_{c}\rangle \;{\hat{\rho }}_{s}-{\mathrm{Tr}}_{b}\left\{{\hat{\rho }}_{c}\;{\hat{H}}_{c}\right\}$ with $\partial_{\beta }Z_{c}=-\langle {\hat{H}}_{c}\rangle \;Z_{c}$. We then obtain

(31) $$\begin{array}{c} T\left(S_{s}-S_{vN}\right)=\beta \;\partial_{\beta }\langle {\hat{H}}_{s}^{*}\rangle +\beta \left[\langle {\hat{H}}_{c}\;{\hat{H}}_{s}^{*}\rangle -\langle {\hat{H}}_{c}\rangle \langle {\hat{H}}_{s}^{*}\rangle \right]\;. \end{array}$$

Thus, part of the difference between the two entropies result from the correlation between the full Hamiltonian ${\hat{H}}_{c}$ and the Hamiltonian of mean force ${\hat{H}}_{s}^{*}$. This correlation will disappear in the vanishing coupling limit because there is no interaction to bridge the system and the bath. We also note that in the same limit, $\langle {\hat{H}}_{s}^{*}\rangle \approx \langle {\hat{H}}_{s}\rangle$ becomes temperature-independent, and both definitions of the entropy turn synonymous.

Since the von Neumann entropy $S_{vN}$ can be used as a measure of entanglement between the system and the bath at zero temperature, we often introduce the quantum mutual information $I_{sb}$ to quantify how they are correlated,

(32) $$I_{sb}=S_{vN}+S_{b}^{\prime }-S_{c}\geq 0\;,$$

where $S_{b}^{\prime }$ is the von Neumann entropy associated with the reduced density matrix ${\hat{\varrho }}_{b}$ of the bath, in contrast to $S_{b}$ we have met earlier. This mutual information can be related to the quantum relative entropy $S({\hat{\rho }}_{c}∥{\hat{\rho }}_{s}⊗{\hat{\varrho }}_{b})$ by

(33) $$S\left({\hat{\rho }}_{c}∥{\hat{\rho }}_{s}⊗{\hat{\varrho }}_{b}\right)={\mathrm{Tr}}_{sb}\left\{{\hat{\rho }}_{c}\ln{\hat{\rho }}_{c}-{\hat{\rho }}_{c}\ln{\hat{\rho }}_{s}⊗{\hat{\varrho }}_{b}\right\}=I_{sb}\;,$$

because ${\hat{\varrho }}_{b}={\mathrm{Tr}}_{s}{\hat{\rho }}_{c}$. On the other hand, Equation (23) imply that the thermodynamic entropy $S_{s}$ is additive $S_{s}+S_{b}=S_{c}$, from which we find

(34) $$I_{sb}=\left(S_{b}^{\prime }-S_{b}\right)+\left(S_{vN}-S_{s}\right)\;.$$

This and (31) provide different perspectives on how the difference between the two system entropies is related to the system-bath entanglement, and how the system-bath coupling has a role in establishing such correlations.

Following the definitions of the internal energy (27) and the entropy (23), the heat capacity of the system still satisfies a familiar relation $C_{s}=-\beta^{2}\;\partial_{\beta }U_{s}=\beta^{2}\;\partial_{\beta }^{2}\ln Z^{*}=-\beta \;\partial_{\beta }S_{s}$. Compared with (18), with the help of (28), we clearly see their difference, caused by different definitions of internal energy, is given by

(35) $$\begin{array}{cc} -\beta^{2}\;\partial_{\beta }\left(U_{s}-\langle {\hat{H}}_{s}\rangle \right) & =-\beta^{2}\;\partial_{\beta }\left[\langle {\hat{H}}_{i}\rangle +\langle {\hat{H}}_{b}\rangle -{\langle {\hat{H}}_{b}\rangle }_{b}\right]. \end{array}$$

## 6. Issues of These Two Approaches: Entropy and Internal Energy

Both equilibrium quantum formulations for thermodynamics at strong coupling are based on plausible assumptions and are mathematically sound. In Approach I outlined in Section 4, one starts with intuitive definitions of the thermodynamics quantities, inspired by traditional thermodynamics for classical systems premised on vanishingly weak coupling between the system and the bath. This leads to modifications in the thermodynamic relations of the relevant thermodynamics quantities. In Approach II delineated in Section 5, one opts to maintain the familiar thermodynamic relations but is compelled to deal with a rather obscure interpretation of the thermodynamic potentials. Although both approaches in the vanishing system-bath coupling limit are compatible, as shown in Section 3.1, they in general entail distinct definitions of the thermodynamic functions. This disparity is amplified with strong system-bath coupling in the deep quantum regime, where quantum coherence plays an increasingly significant role. Thus, even though both approaches possess the same correct classical thermodynamic limit, they are not guaranteed to give unique physical results in the deep quantum regime, even for simple quantum systems, which are areas for interesting further investigations.

To highlight the issues more explicitly, we can apply these two methods to a simple and completely solvable model, namely, a Brownian oscillator linearly but strongly coupled with a large (or infinitely large, as modeled by a scalar field) bath. We will see both approaches at some point, or others that produce ambiguous or paradoxical results. We make a few observations in the following section.

### 6.1. Entropy

(1) It has been discussed in [36,43,44] that the von Neumann entropy $S_{vN}$ will not approach to zero for the finite system-bath coupling in the limit of zero temperature, but the thermodynamic entropy $S_{s}$, defined in Approach II, behaves nicely in the same limit.
(2) It has been shown [49] that if the composite is in a global thermal state, the discrete energy spectrum of the undamped oscillator will become a continuous one with a unique ground level. This supports physics described by the thermodynamic entropy $S_{s}$.
(3) It has been argued [44,45,46] that the entanglement between the system and the bath prevents the von Neumann entropy from approaching zero at zero temperature. Without quantum entanglement between the system and the bath, the lowest energy level of the composite system will be given by the tensor product of the ground state of the unperturbed system and bath, that is, a pure state. In this case, the von Neumann entropy will go to zero as expected, and this is the scenario that occurred in traditional quantum/classical thermodynamics in the vanishing system-bath coupling limit.

### 6.2. Internal Energy

- It has been discussed [37,38,39,40] that the internal energy defined in Approach II can lead to anomalous behavior of the heat capacity in the low temperature limit. When the system, consisting of a quantum oscillator [40] or a free particle [37,38,39] is coupled to a heat bath modeled by a large number of quantum harmonic oscillators, the heat capacity of the system can become negative if the temperature of the bath is sufficiently low. If the internal energy defined in Approach I is used to compute the heat capacity, then it has been shown that the heat capacity remains positive for all nonzero temperatures but vanishes in the zero bath temperature limit, for a system with one harmonic oscillator [37], or a finite number of coupled harmonic oscillators [29]. This discrepancy may result from the fact that the internal energy defined in Approach II contains contributions from the interaction and the bath Hamiltonian.

It seems to imply that in the low-temperature, strong coupling regime, it remains an open question how to properly define the thermodynamic functions; being able to show the well-known behaviors in the classical thermodynamic limit is a necessary but not sufficient condition.

## 7. Quantum Formulation of Jarzynski’s Strong Coupling Thermodynamics

We now provide a quantum formulation of Jarzynski’s classical results [27] but for composite system C kept under thermal equilibrium. The Hamiltonian operator of the composite C=S+B is assumed to take the form

(36) $${\hat{H}}_{c}={\hat{H}}_{s}+{\hat{H}}_{i}+{\hat{H}}_{b}+J\cdot {\hat{A}}_{b}\;,$$

Here in this paper, *J* will be some external, but constant *c*-number drive acting on the bath via a bath operator ${\hat{A}}_{b}$. It can be a constant pressure, as in Jarzynski’s classical formulation, and then ${\hat{A}}_{b}$ will be an operator corresponding to $V_{b}$, conjugated to *P*. However, in general, ${\hat{A}}_{b}$ will be the operator of the quantity conjugated to *J*. This analogy, though formal, provides an alternative route to introduce the operator conjugated to *J*.

If the composite system is in thermal equilibrium at the temperature $\beta^{-1}$, its state is described by the density matrix operator ${\hat{\rho }}_{c}=e^{-\beta \;{\hat{H}}_{c}}/Z_{c}$, where $Z_{c}={\mathrm{Tr}}_{sb}\left\{e^{-\beta \;{\hat{H}}_{c}}\right\}$, a *c*-number, is the partition function of the composite. For later convenience, we also define the corresponding quantities for the bath B when it is coupled to the system S, ${\hat{\rho }}_{b}=e^{-\beta \;({\hat{H}}_{b}+J\cdot {\hat{A}}_{b})}/Z_{b}$ with the bath partition function $Z_{b}={\mathrm{Tr}}_{b}\left\{e^{-\beta \;({\hat{H}}_{b}+J\cdot {\hat{A}}_{b})}\right\}$. We introduce the Hamiltonian operator of mean force ${\hat{H}}_{s}^{*}$ by

(37) $$e^{-\beta \;{\hat{H}}_{s}^{*}}\equiv \frac{1}{Z_{b}}\;{\mathrm{Tr}}_{b}\left\{e^{-\beta \;{\hat{H}}_{c}}\right\}\;,$$

such that the reduced density matrix of the system S takes the form

(38) $$\begin{array}{ccccc} {\hat{\rho }}_{s} & \equiv {\mathrm{Tr}}_{b}{\hat{\rho }}_{c}=\frac{1}{Z_{s}}\;e^{-\beta \;{\hat{H}}_{s}^{*}}\;, & \mathrm{with} & Z_{s} & =\frac{Z_{c}}{Z_{b}}={\mathrm{Tr}}_{s}\left\{e^{-\beta \;{\hat{H}}_{s}^{*}}\right\}\;. \end{array}$$

The quantity $Z_{s}$ can be viewed as an effective partition function of the system S. This is motivated by the observation that, in the absence of coupling between S and B, or in the weak coupling limit, the composite is additive so its partition function is the product of those of the subsystems, i.e., $Z_{c}=Z_{s}Z_{b}$. The difference ${\hat{H}}_{s}^{*}-{\hat{H}}_{s}$ modifies the dynamics of the system S due to its interaction with the bath B.

In fact, by the construction, $e^{-\beta \;H_{s}^{*}}$, once sandwiched by the appropriate states of the system S and expressed in the imaginary-time path integral formalism (for further details regarding the connection with the influence action, please refer to [28,61,62]), is formally $e^{-S_{cg}}$, where $S_{cg}$ is the coarse-grained effective action of the system S, wick-rotated to the imaginary time. Thus formally $\beta ({\hat{H}}_{s}^{*}-{\hat{H}}_{s})$ is equivalent to the influence action in the imaginary time formalism.

Similar to the classical formulations, we may have two different representations of the operator ${\hat{A}}_{s}$ of the system.

### 7.1. “Bare” Representation

In the bare representation, we may define ${\hat{A}}_{s}=\left({\hat{H}}_{s}^{*}-{\hat{H}}_{s}\right)/J$, and the internal energy operator ${\hat{U}}_{s}$ and the enthalpy operator ${\hat{H}}_{s}$, respectively, by ${\hat{U}}_{s}={\hat{H}}_{s}$ and ${\hat{H}}_{s}={\hat{H}}_{s}^{*}$, with expectation values given by $U_{s}={\mathrm{Tr}}_{s}\left\{{\hat{\rho }}_{s}\;{\hat{U}}_{s}\right\}$ and $H_{s}={\mathrm{Tr}}_{s}\left\{{\hat{\rho }}_{s}\;{\hat{H}}_{s}\right\}=U_{s}+J\cdot A_{s}$, corresponding to the internal energy and the enthalpy we are familiar with, respectively. Here $A_{s}={\mathrm{Tr}}_{s}\left\{{\hat{\rho }}_{s}\;{\hat{A}}_{s}\right\}$. The entropy is chosen to be the von Neumann entropy of the system

(39) $$S_{s}={\mathrm{Tr}}_{s}\left\{{\hat{\rho }}_{s}\;\ln{\hat{\rho }}_{s}\right\}=\beta \left(H_{s}-G_{s}\right)\;.$$

The Gibbs free energy $G_{s}$ is defined as $G_{s}=-\beta^{-1}\ln Z_{s}$. These definitions are in exact parallel to those in the classical formulation [27].

### 7.2. “Partial Molar” Representation

In contrast to the bare representation, we can alternatively define the operator ${\hat{A}}_{s}$ of the system S that corresponds to ${\hat{A}}_{b}$ of the bath B by ${\hat{A}}_{s}=\partial \left({\hat{H}}_{s}^{*}-{\hat{H}}_{s}\right)/\partial J=\partial {\hat{H}}_{s}^{*}/\partial J$. The last equality results from the fact that ${\hat{H}}_{s}$ has no dependence on the external parameter *J*. Owing to the non-commutativity of operators, the micro-physics interpretation of the operator ${\hat{A}}_{s}$ is not so transparent. We first focus on its quantum expectation value $A_{s}$

(40) $$\begin{array}{c} A_{s}={\mathrm{Tr}}_{s}\left\{{\hat{\rho }}_{s}\;{\hat{A}}_{s}\right\}=\frac{1}{Z_{s}}{\mathrm{Tr}}_{s}\left\{e^{-\beta \;{\hat{H}}_{s}^{*}}\;\frac{\partial {\hat{H}}_{s}^{*}}{\partial J}\right\}\;. \end{array}$$

As stressed earlier, since the operator does not commute with its derivative, care must be taken when we move the derivative around in an operator expression. However, from (A7), we learn that the righthand side of (40) can be identified as

(41) $${\mathrm{Tr}}_{s}\left\{e^{-\beta \;{\hat{H}}_{s}^{*}}\;\frac{\partial {\hat{H}}_{s}^{*}}{\partial J}\right\}=-\beta^{-1}\;\frac{\partial }{\partial J}{\mathrm{Tr}}_{s}\left\{e^{-\beta \;{\hat{H}}_{s}^{*}}\right\}\;,$$

and thus we have $A_{s}=-\beta^{-1}\;\partial \ln Z_{s}/\partial J$. The advantage of this expression is that the observation of $Z_{s}=Z_{c}/Z_{b}$ enables us to write $A_{s}$ as $A_{s}=A_{c}-A_{b}$, if we have defined the corresponding expectation values for the composite C and the bath B by $A_{c}=-\beta^{-1}\;\partial \ln Z_{c}/\partial J$ and $A_{b}=-\beta^{-1}\;\partial \ln Z_{b}/\partial J$. In particular we can check that $A_{b}$ indeed is the expectation value of the operator ${\hat{A}}_{b}$, that is, $A_{b}={\mathrm{Tr}}_{b}\left\{{\hat{\rho }}_{b}\;{\hat{A}}_{b}\;\right\}={\mathrm{Tr}}_{sb}\left\{{\hat{\rho }}_{b}\;{\hat{A}}_{b}\;\right\}$. The latter expression can nicely bridge with $A_{c}$ for the composite, $A_{c}={\mathrm{Tr}}_{sb}\left\{\hat{\rho }\;{\hat{A}}_{b}\;\right\}$. Thus the expectation value A is additive. Its value for the combined systems is equal to the sum of those of the subsystems, $A_{c}=A_{s}+A_{b}$. In fact, this additive property holds for all the thermodynamics potentials introduced afterwards. This is a nice feature in Jarzynski’s partial molar representation or in Seifert’s approach.

From this aspect, we can interpret $A_{s}$ as the change of $A_{b}$ due to the intervention of the system S. For example, consider a photon gas inside a cavity box, one side of which is a movable classical mirror and is exerted by a constant pressure. Assume originally the photon gas and the mirror are in thermal equilibrium. In this cavity we now place a Brownian charged oscillator and maintain the new composite system in thermal equilibrium at the same temperature and the same pressure (The equilibration process in this example can be awfully complicated if we mind the subtleties regarding whether the photon gas can ever reach thermal equilibrium in a cavity whose walls are perfectly reflective and so on. For the present argument, we assume equilibration is possible and there is no leakage of the photons). Then we should note that there is a minute change in the mean position of the mirror before and after the Brownian charged oscillator is placed into the cavity. This change can also be translated to an effective or dynamical size of the charged oscillator due to its interaction with the photon gas, and thus is accounted for in $A_{s}$ when *J* is identified as the constant pressure applied to the wall.

From this example, it is tempting to identify $J\cdot {\hat{A}}_{s}$ as some quantum work operator (its value depends on the interaction between the system and the bath and when this interaction is switched on. It is thus path-dependent in the parameter space of the coupling constant). Alternatively we may view it or its expectation as some additional “energy content” of the system S due to its interaction with the bath when the composite is acted upon by an external agent *J*, since ${\hat{A}}_{s}$ is related to ${\hat{H}}_{s}^{*}-{\hat{H}}_{s}$ [63,64]. Inspired by this observation and taking the hint from the definition of $A_{s}$, we introduce the enthalpy of the system S by

(42) $$H_{s}\equiv -\frac{\partial }{\partial \beta }\ln Z_{s}=H_{c}-H_{b}\;,$$

where we have identified the enthalpies of the composite C=S+B and the bath B as $H_{c}=-\partial \ln Z_{c}/\partial \beta$ and $H_{b}=-\partial \ln Z_{b}/\partial \beta$. We may rewrite them as $H_{c}=\langle {\hat{H}}_{s}\rangle +\langle {\hat{H}}_{i}\rangle +\langle {\hat{H}}_{b}\rangle +J\cdot A_{c}$ and $H_{b}={\langle {\hat{H}}_{b}\rangle }_{b}+J\cdot A_{b}$. It implies that (1) the system enthalpy can be decomposed as

(43) $$\begin{array}{cc} H_{s}=H_{c}-H_{b} & =\left[\langle {\hat{H}}_{s}\rangle +\langle {\hat{H}}_{i}\rangle +\langle {\hat{H}}_{b}\rangle -{\langle {\hat{H}}_{b}\rangle }_{b}\right]+J\cdot A_{s}\;, \end{array}$$

and (2) the internal energy $U_{s}$ of the system S can be consistently inferred as

(44) $$U_{s}=\langle {\hat{H}}_{s}\rangle +\langle {\hat{H}}_{i}\rangle +\left[\langle {\hat{H}}_{b}\rangle -{\langle {\hat{H}}_{b}\rangle }_{b}\right]\;.$$

This is exactly the same internal energy obtained in Seifert’s approach in the equilibrium setting. We can define the internal energy of the composite system and of the bath by $U_{c}=\langle {\hat{H}}_{s}\rangle +\langle {\hat{H}}_{i}\rangle +\langle {\hat{H}}_{b}\rangle$ and $U_{b}={\langle {\hat{H}}_{b}\rangle }_{b}$, and then we may conclude $U_{s}=U_{c}-U_{b}$. Thus the internal energy $U_{s}$ also includes contributions that naïvely we will not ordinarily attribute to the system, such as $\langle {\hat{H}}_{b}\rangle -{\langle {\hat{H}}_{b}\rangle }_{b}$. Doing so will complicate the physical connotation of the internal energy of the system.

Up to this moment, we essentially write the thermodynamic quantities by the quantum expectation value and in terms of the partition functions. Thus it is appropriate to introduce the Gibbs free energies of the composite C, the system S, and the bath B by $G_{a}=-\beta^{-1}\ln Z_{a}$, where a=c, *s* and *b*, and they obey the additive property of the Gibbs energy, $G_{c}=G_{s}+G_{b}$. Furthermore, in the composite, we note that

(45) $$\begin{array}{c} \beta \left(H_{c}-G_{c}\right)=\beta^{2}\frac{\partial G_{c}}{\partial \beta }=-{\mathrm{Tr}}_{sb}\left\{{\hat{\rho }}_{c}\;\ln{\hat{\rho }}_{c}\right\}\;. \end{array}$$

From (45), we can consistently define the entropy S of the composite by $S_{c}=\beta \left(H_{c}-G_{c}\right)$ and, similarly, the entropy $S_{b}$ of the bath:

(46) $$S_{b}=\beta \left(H_{b}-G_{b}\right)=\beta^{2}\;\frac{\partial G_{b}}{\partial \beta }=-{\mathrm{Tr}}_{b}\left\{{\hat{\rho }}_{b}\;\ln{\hat{\rho }}_{b}\right\}\;.$$

The additive property of the free energy and the enthalpy implies that the entropy $S_{s}$ of the system in this representation is also additive, $S_{s}=S_{c}-S_{b}$, and is given by

(47) $$S_{s}=\beta \left(H_{s}-G_{s}\right)=\beta^{2}\;\frac{\partial G_{s}}{\partial \beta }=-{\mathrm{Tr}}_{sb}\left\{{\hat{\rho }}_{c}\;\ln{\hat{\rho }}_{c}\right\}+{\mathrm{Tr}}_{b}\left\{{\hat{\rho }}_{b}\;\ln{\hat{\rho }}_{b}\right\}\neq -{\mathrm{Tr}}_{s}\left\{{\hat{\rho }}_{s}\;\ln{\hat{\rho }}_{s}\right\}\;,$$

Note it is not equal to the von Neumann entropy, which is defined as the entropy of the system in the “bare” representation.

### 7.3. Operator Forms of the Thermodynamic Functions

In trying to formulate a set of laws to describe the thermodynamics of a quantum system (even the existence of such a theory, under certain appropriate conditions, is not a matter of presumption or prescription, but by demonstration and proof) it would be most convenient if we could define operators of the thermodynamic functions in such a way that the quantum expectation values of those operators give the familiar expressions for the thermodynamic functions. As we see it, this is the paramount challenge in the formal establishment of quantum thermodynamics as a viable theory. The laws of thermodynamics have been understood in terms of the mean values of the relevant operator quantities. For a system where the fluctuations of the thermodynamic functions become comparable to the corresponding mean values, the thermodynamic laws governing the mean values need be supplanted by laws governing their quantum fluctuations or higher order quantum correlations. A case in point for classical systems where fluctuations are as important as the mean values is near the critical point of the system. The truly quantum properties would impact on the quantum thermodynamics for small quantum systems in the regimes of strong couplings to its environment, and at low temperatures, where quantum coherence effects take center stage. Having the operator forms of these thermodynamic potentials allows us to calculate the higher-order quantum correlations of those quantities existent in larger fluctuations.

In the following sections, we will attempt to identify the operator form of the thermodynamic function for the reduced system.

#### 7.3.1. Enthalpy and Energy Operators: Caution

In fact, we may deduce the operator form of the quantities introduced earlier. For example, we may intuitively define the enthalpy operator $\hat{H}$ of the composite by ${\hat{H}}_{c}={\hat{H}}_{s}+{\hat{H}}_{i}+{\hat{H}}_{b}+J\cdot {\hat{A}}_{b}$, and then it is clear to see that the expectation value $H_{c}$ is related to this operator by $H_{c}={\mathrm{Tr}}_{sb}\left\{{\hat{\rho }}_{c}\;{\hat{H}}_{c}\right\}=\langle {\hat{H}}_{c}\rangle$. Likewise, the enthalpy operator ${\hat{H}}_{b}$ of the bath B can be defined by ${\hat{H}}_{b}={\hat{H}}_{b}+J\cdot {\hat{A}}_{b}$, and its expectation value gives $H_{b}={\mathrm{Tr}}_{b}\left\{{\hat{\rho }}_{b}\;{\hat{H}}_{b}\right\}={\langle {\hat{H}}_{b}\rangle }_{b}$. Moreover, the internal energy operator ${\hat{U}}_{c}$ of the composite system and the expectation value can be chosen such that ${\hat{U}}_{c}={\hat{H}}_{s}+{\hat{H}}_{i}+{\hat{H}}_{b}$ such that $U_{c}={\mathrm{Tr}}_{sb}\left\{{\hat{\rho }}_{c}\;{\hat{U}}_{c}\right\}=\langle {\hat{U}}_{c}\rangle$. For the bath, the internal energy operator ${\hat{U}}_{b}$ is, intuitively, ${\hat{U}}_{b}={\hat{H}}_{b}$ with expectation values $U_{b}={\mathrm{Tr}}_{b}\left\{{\hat{\rho }}_{b}\;{\hat{U}}_{b}\right\}={\langle {\hat{U}}_{b}\rangle }_{b}$ that is consistent with the expressions of the internal energy discussed earlier.

Despite their intuitively appealing appearances, these operator forms of the enthalpies and internal energies are not very useful. Inadvertent use of them may result in errors. For example, we cannot define the enthalpy operator of system S simply by the difference of ${\hat{H}}_{c}$ and ${\hat{H}}_{b}$, since

(48) $$\begin{array}{c} {\hat{H}}_{s}\overset{?}{=}{\hat{H}}_{c}-{\hat{H}}_{b}={\hat{H}}_{s}+{\hat{H}}_{i}\;. \end{array}$$

This result in (48) is nonsensical because (1) the righthand side still explicitly depends on the bath degree of freedom; (2) we cannot take its trace with respect to the state of the system, ${\hat{\rho }}_{s}$; and thus (3) the expectation value will not be $H_{s}$. This is because the operators defined this way act on Hilbert spaces different from that of ${\hat{\rho }}_{s}$; ${\hat{H}}_{c}$ is an operator in the Hilbert space of the composite while ${\hat{H}}_{b}$ is an operator in the Hilbert space of the bath. Neither operator acts exclusively in the Hilbert space of the system. Thus, extreme care is needed when manipulating the operator forms of the thermodynamical potentials. What one needs to do is to seek the local forms of these operators, i.e., operators which act only on the Hilbert space of the system. This can be done in parallel to Jarzynski’s classical formulation.

#### 7.3.2. System Enthalpy Operator: Approved

We first inspect the internal energy operator. Since the averaged internal energy of the composite system is given by $U_{c}={\mathrm{Tr}}_{sb}\left\{e^{-\beta \;{\hat{H}}_{c}}\;\left({\hat{H}}_{s}+{\hat{H}}_{i}+{\hat{H}}_{b}\right)\right\}/Z_{c}$, we can rewrite the expressions inside the trace into

(49) $$\begin{array}{cc} U_{c} & ={\mathrm{Tr}}_{s}\left\{{\hat{\rho }}_{s}\;{\hat{Z}}_{i}^{-1}{\mathrm{Tr}}_{b}\left[e^{+\beta \;{\hat{H}}_{s}}e^{-\beta \;{\hat{H}}_{c}}\;\left({\hat{H}}_{s}+{\hat{H}}_{i}+{\hat{H}}_{b}\right)\right]\right\}\;, \end{array}$$

in a way analogous to Jarzynksi’s classical formulation. Here we have used the fact that $Z_{c}=Z_{s}Z_{b}$ and the identity for the operator ${\hat{Z}}_{i}$

$$\begin{array}{ccccc} {\hat{Z}}_{i} & \equiv e^{+\beta \;{\hat{H}}_{s}}\;{\mathrm{Tr}}_{b}\left\{e^{-\beta \;\left({\hat{H}}_{s}+{\hat{H}}_{i}+{\hat{H}}_{b}+J\cdot {\hat{A}}_{b}\right)}\right\}=Z_{b}\;e^{+\beta \;{\hat{H}}_{s}}e^{-\beta \;{\hat{H}}_{s}^{*}}\;, & \Leftrightarrow & \frac{e^{+\beta \;{\hat{H}}_{s}^{*}}}{Z_{b}} & ={\hat{Z}}_{i}^{-1}\;e^{+\beta \;{\hat{H}}_{s}}\;. \end{array}$$

If we define an internal energy operator ${\hat{U}}_{i}$ by

(50) $${\hat{U}}_{i}={\hat{Z}}_{i}^{-1}{\mathrm{Tr}}_{b}\left[e^{+\beta \;{\hat{H}}_{s}}e^{-\beta \;{\hat{H}}_{c}}\;\left({\hat{H}}_{s}+{\hat{H}}_{i}+{\hat{H}}_{b}\right)\right]=Z_{b}^{-1}e^{+\beta \;{\hat{H}}_{s}^{*}}{\mathrm{Tr}}_{b}\left[e^{-\beta \;{\hat{H}}_{c}}\;\left({\hat{H}}_{s}+{\hat{H}}_{i}+{\hat{H}}_{b}\right)\right]\;,$$

then we obtain a new representation of $U_{c}$

(51) $$U_{c}={\mathrm{Tr}}_{s}\left\{{\hat{\rho }}_{s}\;{\hat{U}}_{i}\right\}\;.$$

Equation (50) is an operator expression of the internal energy of the composite system, on account of the non-commutativity of the operators, but its expectation value is taken with respect to the system’s density matrix ${\hat{\rho }}_{s}$. With the help of (A9), this is equivalent to Equation (28) of [26] in the J=0 case. In addition, we note that ${\hat{Z}}_{i}$ is an operator, not a *c* number. Since ${\mathrm{Tr}}_{s}{\hat{\rho }}_{s}=1$, we may define the operator ${\hat{U}}_{s}$ by

(52) $${\hat{U}}_{s}={\hat{U}}_{i}-U_{b}\;,$$

such that ${\mathrm{Tr}}_{s}\left\{{\hat{\rho }}_{s}\;{\hat{U}}_{s}\right\}={\mathrm{Tr}}_{s}\left\{{\hat{\rho }}_{s}\;{\hat{U}}_{i}\right\}-{\mathrm{Tr}}_{s}\left\{{\hat{\rho }}_{s}\;U_{b}\right\}=U_{c}-U_{b}=U_{s}$. The advantage of (50), (52) is that, unlike those introduced in the previous subsection, they are all operators in the Hilbert space of the system S. Indeed, using the identity operator ${\hat{I}}_{s}$ in the Hilbert space of the system S we can also define ${\hat{U}}_{b}$ as ${\hat{U}}_{b}=U_{b}\;{\hat{I}}_{s}$.

In the same fashion, we may rewrite $A_{c}$ by

(53) $$\begin{array}{cc} A_{c}={\mathrm{Tr}}_{s}\left\{\frac{1}{Z}{\mathrm{Tr}}_{s}\left[e^{-\beta \;\hat{H}}\;{\hat{A}}_{b}\right]\right\} & ={\mathrm{Tr}}_{s}\left\{{\hat{\rho }}_{s}\;{\hat{Z}}_{i}^{-1}{\mathrm{Tr}}_{b}\left[e^{+\beta \;{\hat{H}}_{s}}e^{-\beta \;\hat{H}}\;{\hat{A}}_{b}\right]\right\}\;. \end{array}$$

Thus we can define

(54) $${\hat{A}}_{i}={\hat{Z}}_{i}^{-1}{\mathrm{Tr}}_{b}\left[e^{+\beta \;{\hat{H}}_{s}}e^{-\beta \;\hat{H}}\;{\hat{A}}_{b}\right]\;,$$

so that $A_{c}={\mathrm{Tr}}_{s}\left\{{\hat{\rho }}_{s}\;{\hat{A}}_{i}\right\}$. We then can have a local form for the ${\hat{A}}_{s}$ given by ${\hat{A}}_{s}={\hat{A}}_{i}-{\hat{A}}_{b}$ in close resemblance to its classical expression in [27], if we re-define ${\hat{A}}_{b}$ as ${\hat{A}}_{b}=A_{b}\;{\hat{I}}_{s}$. The expectation value of ${\hat{A}}_{s}$ is then given by ${\mathrm{Tr}}_{s}\left\{{\hat{\rho }}_{s}\;{\hat{A}}_{s}\right\}={\mathrm{Tr}}_{s}\left\{{\hat{\rho }}_{s}\;{\hat{A}}_{i}\right\}-A_{b}=A_{c}-A_{b}=A_{s}$.

Now we proceed with constructing a local form of the enthalpy operator of the system. From (52) and the definition of the operator ${\hat{A}}_{s}$, we claim that the local form ${\hat{H}}_{s}$ is

(55) $${\hat{H}}_{s}={\hat{U}}_{s}+J\cdot {\hat{A}}_{s}\;.$$

We can straightforwardly show that ${\mathrm{Tr}}_{s}\left\{{\hat{\rho }}_{s}\;{\hat{H}}_{s}\right\}={\mathrm{Tr}}_{s}\left\{{\hat{\rho }}_{s}\;{\hat{U}}_{s}\right\}+J\cdot {\mathrm{Tr}}_{s}\left\{{\hat{\rho }}_{s}\;{\hat{A}}_{s}\right\}=U_{s}+J\cdot A_{s}$. Thus we have succeeded in constructing the operators that correspond to $A_{s}$, $U_{s}$, $H_{s}$ in forms local in the Hilbert space of the system S. However, as can be seen from their expressions, their meanings are not transparent a priori. They are determined a posteriori because we would like their expectation values to take certain forms. This can pose a question about the uniqueness of these operators (A similar issue is also raised in [58] for the classical formulation. However, in this context it is not clear whether this ambiguity can be fixed by calculating the cumulants associated with these operators. If there exist physical, measurable observables that correspond to the expectation values of the moments of these operators, then one may entertain the possibility of using them to uniquely determine these operators.). At least for a given reduced density matrix ${\hat{\rho }}_{s}$ of the system, we can always attach a system operator ${\hat{\Lambda }}_{s}$ that satisfies ${\mathrm{Tr}}_{s}{\hat{\rho }}_{s}{\hat{\Lambda }}_{s}=0$ to the definitions of those local operators, that is, any system operator that has a zero mean. The choice of ${\hat{\Lambda }}_{s}$ may not be unique in the sense that in the basis {|n〉} that diagonalizes ${\hat{\rho }}_{s}$, we can write ${\mathrm{Tr}}_{s}\left\{{\hat{\rho }}_{s}{\hat{\Lambda }}_{s}\right\}=0$ as

(56) $${\mathrm{Tr}}_{s}\left\{{\hat{\rho }}_{s}{\hat{\Lambda }}_{s}\right\}=\sum_{m,n}\langle n|{\hat{\rho }}_{s}\left|m\rangle \langle m\right|{\hat{\Lambda }}_{s}|n\rangle =\sum_{n}{\left({\hat{\rho }}_{s}\right)}_{nn}{\left({\hat{\Lambda }}_{s}\right)}_{nn}=0\;.$$

It says that the vectors that are respectively composed of the diagonal elements of ${\hat{\rho }}_{s}$ and ${\hat{\Lambda }}_{s}$ are orthogonal, but it does not place any restriction on the off-diagonal elements of ${\hat{\Lambda }}_{s}$ on this basis.

## 8. Conclusions

### 8.1. Summary

In this paper we provide quantum formulations of equilibrium thermodynamic functions and their relations for Jarzynski’s classical thermodynamics at strong coupling [27] without the consideration of heat and work. The combined system + environment, called the composite, is assumed initially to be in a global thermal state. In such a configuration, even though the interaction between the system and the bath is non-negligible, the partition function of the composite is well defined. This facilitates the introduction of thermodynamic potentials in a way similar to the traditional vanishing-coupling thermodynamics. Such a configuration allows for the introduction of enthalpy by introducing an external agent (keeping at constant pressure in Jarzynski’s case) acting on the conjugate bath operator. The effect can be represented by an equivalent effect on the system, which then appears in the expression of the enthalpy of the system. There are two ways to capture this effect, called the “bare” and “partial molar” representations by Jarzynski. We have worked out a quantum formulation for each of these two representations of Jarzynski’s classical thermodynamics. In addition, we attempt to identify the operator forms of the thermodynamic functions, which can be potentially useful in directly addressing the effects of quantum fluctuations on thermodynamics at strong coupling, where the fluctuations can reach the same order of magnitude as the corresponding mean values.

### 8.2. Issues

We mention two outstanding issues of the two representations or approaches, namely, internal energy and entropy of the system at strong coupling in the global thermal state. When the quantum versions of these two approaches are applied to a small quantum system that strongly couples with a low-temperature bath, some nonintuitive results have been reported in the literature [29,36,37,38,39,40,43,44]. If the von Neumann entropy is adopted as the system entropy, then when the system and the bath are entangled, this entropy will not approach zero for a simple system such as a harmonic oscillator in the zero temperature bath, contradicting the result in [49], where it has been shown that the ground state of such a composite system is non-degenerate in general, thus implying vanishing entropy at zero temperature. On the other hand, if the system’s internal energy is defined as the derivative of the partition function of the system, the heat capacity derived therefrom can take on negative values in the low temperature regime when the system consists of free particles or coupled harmonic oscillators. This anomalous behavior, not seen when the internal energy defined as the expectation value of the system Hamiltonian, may be traced to an excessive inclusion of the interaction and the bath contributions, as shown in (44), in the definition of the internal energy of the system.

### 8.3. Further Developments

To complete a theory for equilibrium quantum thermodynamics, we need to include the considerations of heat and work. For understanding quantum work, the physical meaning of the operator ${\hat{A}}_{s}$ conjugate to *J* is a key issue. At the next level of investigation, reaching out to nonequilibrium conditions, also at strong coupling, we posit that the operator ${\hat{\Delta }}_{s}$, introduced by Gelin and Thoss, and the Hamiltonian operator of mean force can be related to the influence action or the coarse-grained effective action [28,61,62] of the system when they are sandwiched by the states of the system and formulated in the finite temperature imaginary-time path integral method. This and an earlier observation we made for the partition function point out a way to extend the present equilibrium quantum thermodynamics at strong coupling for a closed system in a global thermal state to a nonequilibrium setting, by employing the real-time closed-time-path formalism used in [28,29,61,62]. This is the goal of our next paper in this series [65].

## Appendix A. Handling Operator Products in Quantum Thermodynamics

In deriving various thermodynamic relations in the context of quantum thermodynamics, we often come up with expressions involving exponential of the sum of two operators, say $\hat{\lambda }$ and $\hat{\mu }$. Unlike its *c*-number counterpart, in general such an exponential cannot be written as a product of exponential of the respective operators, that is,

(A1) $$e^{\hat{\lambda }+\hat{\mu }}\overset{?}{=}e^{\hat{\lambda }}\;e^{\hat{\mu }}\;,$$

because these two operators $\hat{\lambda }$, $\hat{\mu }$ may not commute. From Baker-Campbell-Haussdorff formulas, the righthand side of (A1) in fact is given by

(A2) $$e^{\hat{\lambda }}e^{\hat{\mu }}=\exp\left(\hat{\lambda }+\hat{\mu }+\frac{1}{2!}\;\left[\hat{\lambda },\hat{\mu }\right]+\frac{1}{3!}\;\left\{\frac{1}{2}\;\left[\left[\hat{\lambda },\hat{\mu }\right],\hat{\mu }\right]+\frac{1}{2}\;\left[\hat{\lambda },\left[\hat{\lambda },\hat{\mu }\right]\right]\right\}+\cdots \right)\;,$$

for any two operators $\hat{\lambda }$, $\hat{\mu }$. However, in the special case that $[\hat{\lambda },\hat{\mu }]=0$, the equality in (A1) indeed is valid. The other useful expression in the Baker-Campbell-Hausdorff formulas is

(A3) $$e^{\hat{\lambda }}\;\hat{\mu }\;e^{-\hat{\lambda }}=\hat{\mu }+\left[\hat{\lambda },\hat{\mu }\right]+\frac{1}{2}\;\left[\hat{\lambda },\left[\hat{\lambda },\hat{\mu }\right]\right]+\cdots \;.$$

This is particularly useful in deriving the unitary transformation of $\hat{\mu }$ by the operator $e^{\hat{\lambda }}$.

We also often come to a situation that we need to take a derivative of an exponential of the operator. This is less straightforward than is expected due to the fact that the operator in the exponent may not commute with its own derivative. For example, consider an operator $\hat{O}\left(\chi \right)$ of the form $\hat{O}\left(\chi \right)=\alpha \left(\chi \right)\;\hat{X}+\beta \left(\chi \right)\;\hat{P}$, with α, β being functions of χ, but the operators $\hat{X}$, $\hat{P}$ of the canonical variables have no explicit χ dependence. We immediately see the trivial result $[\hat{O}\left(\chi \right),\;\hat{O}\left(\chi \right)]=0$, but $\left[\hat{O}\left(\chi \right),\partial_{\chi }\hat{O}\left(\chi \right)\right]=\left(\alpha \dot{\beta }-\dot{\alpha }\beta \right)\;\left[\hat{X},\hat{P}\right]\neq 0$, where the overhead dot represents the derivative with respect to χ. This introduces complications in taking the derivative of, say, $e^{-\hat{O}\left(\chi \right)}$ with respect to χ. If we realize an operator function in terms of its Taylor’s expansion, then

(A4) $$e^{-\hat{O}\left(\chi \right)}=\sum_{k=0}^{\infty }\frac{{\left(-1\right)}^{k}}{k!}\;{\hat{O}}^{k}\left(\chi \right)\;.$$

Taking the derivative of (A4) with respective to χ yields

(A5) $$\begin{array}{c} \;\partial_{\chi }e^{-\hat{O}}\\ =-\sum_{k=1}^{\infty }\frac{{\left(-1\right)}^{k-1}}{(k-1)!}\;\left\{\frac{1}{k}\;\left[\left(\partial_{\chi }\hat{O}\right)\underset{(k-1)\;\mathrm{terms}}{\underset{︸}{\hat{O}\cdots \cdots \hat{O}}}+\hat{O}\left(\partial_{\chi }\hat{O}\right)\underset{(k-2)\;\mathrm{terms}}{\underset{︸}{\hat{O}\cdots \cdots \hat{O}}}+\cdots \cdots +\underset{(k-1)\;\mathrm{terms}}{\underset{︸}{\hat{O}\cdots \cdots \hat{O}}}\left(\partial_{\chi }\hat{O}\right)\right]\right\}\\ =-{\left[\left(\partial_{\chi }\hat{O}\right)\;e^{-\hat{O}}\right]}_{\mathrm{sym}}\;, \end{array}$$

where we define the symmetrized product ${\left({\hat{O}}_{1}{\hat{O}}_{2}\cdots {\hat{O}}_{k}\right)}_{\mathrm{sym}}$ as a generalization of the anti-commutator by

(A6) $${\left({\hat{O}}_{1}{\hat{O}}_{2}\cdots {\hat{O}}_{k}\right)}_{\mathrm{sym}}=\frac{1}{\#\mathrm{of}\;\mathrm{perm}.}\sum_{\#\mathrm{of}\;\mathrm{perm}.}{\hat{O}}_{\sigma_{1}}{\hat{O}}_{\sigma_{2}}\cdots {\hat{O}}_{\sigma_{k}}\;,$$

with σ being the permutations of 1, 2, …, *k*. Thus the expression, say, $\left(\partial_{\beta }{\hat{H}}_{s}^{*}\right)\;e^{-\beta \;{\hat{H}}_{s}^{*}}$, and similar derivative expressions will be understood in this manner as a symmetrized product of $\partial_{\beta }{\hat{H}}_{s}^{*}$ and the Taylor-expanded $e^{-\beta \;{\hat{H}}_{s}^{*}}$, shown in (A5).

However, if the derivative like (A5) is taken within a trace, then the complicated expression (A5) will reduce to a simple form

(A7) $$\begin{array}{c} \mathrm{Tr}\left\{\partial_{\chi }e^{-\hat{O}}\right\}=-\sum_{k=1}^{\infty }\frac{{\left(-1\right)}^{k-1}}{(k-1)!}\;\mathrm{Tr}\left\{\left(\partial_{\chi }\hat{O}\right)\;{\hat{O}}^{k-1}\right\}=-\mathrm{Tr}\left\{\left(\partial_{\chi }\hat{O}\right)\;e^{-\hat{O}}\right\}\;, \end{array}$$

due to the cyclic property of the trace formula. Hence in general we have $\partial_{\chi }e^{-\hat{O}}=-{\left[\left(\partial_{\lambda }\hat{O}\right)\;e^{-\hat{O}}\right]}_{\mathrm{sym}}$, but its trace gives

(A8) $$\mathrm{Tr}\left\{\partial_{\chi }e^{-\hat{O}}\right\}=-\mathrm{Tr}\left\{{\left[\left(\partial_{\lambda }\hat{O}\right)\;e^{-\hat{O}}\right]}_{\mathrm{sym}}\right\}=-\mathrm{Tr}\left\{\left(\partial_{\chi }\hat{O}\right)\;e^{-\hat{O}}\right\}\;,$$

as if the operator $\hat{O}$ is a *c*-number. Note here we have assumed the traces applied in (A7) and (A8) are not a partial trace; otherwise the same symmetrization procedure is still needed.

A special case of (A7) is $\partial_{\chi }e^{-\chi \;\hat{O}}$, in which $\hat{O}$ has no explicit dependence on χ. Then it is straightforward to perform the differentiation, and since $[\hat{O},e^{-\chi \;\hat{O}}]=0$, we obtain

(A9) $$\partial_{\chi }e^{-\chi \;\hat{O}}=-\hat{O}\;e^{-\chi \;\hat{O}}\;.$$

Next we give an explicit application of (A7). In particular, we focus on the expression

(A10) $$\begin{array}{c} \;-\frac{1}{Z_{c}}\frac{\partial }{\partial \beta }{\mathrm{Tr}}_{s}e^{-\beta ({\hat{H}}_{s}+{\hat{\Delta }}_{s})}\;,\;\;\mathrm{with}\;\;Z_{c}={\mathrm{Tr}}_{s}e^{-\beta ({\hat{H}}_{s}+{\hat{\Delta }}_{s})}\;. \end{array}$$

Carrying out the differentiation of (A10) gives

(A11) $$\begin{array}{c} =\frac{1}{Z_{c}}{\mathrm{Tr}}_{s}\left\{\left({\hat{H}}_{s}+{\hat{\Delta }}_{s}+\beta \;\partial_{\beta }{\hat{\Delta }}_{s}\right)e^{-\beta ({\hat{H}}_{s}+{\hat{\Delta }}_{s})}\right\}=\langle {\hat{H}}_{s}\rangle +\langle {\hat{\Delta }}_{s}\rangle +\beta \;\langle \partial_{\beta }{\hat{\Delta }}_{s}\rangle \;. \end{array}$$

The first two terms after the first equal sign in (A11) is the consequence of (A9), while the third term results from (A8) due to the trace.
